# Revisiting diabetes risk of olanzapine versus aripiprazole in serious mental illness care

**DOI:** 10.1192/bjo.2024.727

**Published:** 2024-08-08

**Authors:** Denis Agniel, Sharon-Lise T. Normand, John W. Newcomer, Katya Zelevinsky, Jason Poulos, Jeannette Tsuei, Marcela Horvitz-Lennon

**Affiliations:** RAND Corporation, Santa Monica, California, USA; Department of Health Care Policy, Harvard Medical School, Boston, Massachusetts, USA; and Department of Biostatistics, Harvard TH Chan School of Public Health, Boston, Massachusetts, USA; Department of Psychiatry, Washington University School of Medicine, St Louis, Missouri, USA; and Thriving Mind South Florida, Miami, Florida, USA; Department of Health Care Policy, Harvard Medical School, Boston, Massachusetts, USA; RAND Corporation, Boston, Massachusetts, USA; and Department of Psychiatry, Cambridge Health Alliance and Harvard Medical School, Cambridge, Massachusetts, USA

**Keywords:** Antipsychotics, causal inference, machine learning methods

## Abstract

**Background:**

Exposure to second-generation antipsychotics (SGAs) carries a risk of type 2 diabetes, but questions remain about the diabetogenic effects of SGAs.

**Aims:**

To assess the diabetes risk associated with two frequently used SGAs.

**Method:**

This was a retrospective cohort study of adults with schizophrenia, bipolar I disorder or severe major depressive disorder (MDD) exposed during 2008–2013 to continuous monotherapy with aripiprazole or olanzapine for up to 24 months, with no pre-period exposure to other antipsychotics. Newly diagnosed type 2 diabetes was quantified with targeted minimum loss-based estimation; risk was summarised as the restricted mean survival time (RMST), the average number of diabetes-free months. Sensitivity analyses were used to evaluate potential confounding by indication.

**Results:**

Aripiprazole-treated patients had fewer diabetes-free months compared with olanzapine-treated patients. RMSTs were longer in olanzapine-treated patients, by 0.25 months [95% CI: 0.14, 0.36], 0.16 months [0.02, 0.31] and 0.22 months [0.01, 0.44] among patients with schizophrenia, bipolar I disorder and severe MDD, respectively. Although some sensitivity analyses suggest a risk of unobserved confounding, E-values indicate that this risk is not severe.

**Conclusions:**

Using robust methods and accounting for exposure duration effects, we found a slightly higher risk of type 2 diabetes associated with aripiprazole compared with olanzapine monotherapy regardless of diagnosis. If this result was subject to unmeasured selection despite our methods, it would suggest clinician success in identifying olanzapine candidates with low diabetes risk. Confirmatory research is needed, but this insight suggests a potentially larger role for olanzapine in the treatment of well-selected patients, particularly for those with schizophrenia, given the drug's effectiveness advantage among them.

Non-clozapine second-generation antipsychotics (SGAs) are routinely used as first-line agents for schizophrenia and, increasingly, for bipolar I disorder^1^ and treatment-resistant major depressive disorder (MDD),^2^ the serious mental illnesses (SMI) for which all or some SGAs have US Food and Drug Administration (FDA) approval. Although antipsychotics have been associated with adverse effects varying in persistence and severity, cardiometabolic morbidity, including type 2 diabetes and its risk factors (weight gain and metabolic dysregulation), ranks among the top safety concerns for SGAs.^3^ Current evidence suggests that among commonly prescribed SGAs, this risk is higher for olanzapine and lower for aripiprazole, with risperidone and quetiapine conferring an intermediate risk.^4^ The association between antipsychotics and type 2 diabetes, hereafter diabetes, first hypothesised in the 1950s,^5^ became a focus of significant concern in the early 2000s^6^ at a time when non-clozapine SGAs had overtaken the US antipsychotic market. Diabetes is at least twice as prevalent among people with SMI compared with those without SMI and also more severe,^7^ its significance heightened by its association with cardiovascular disease,^8^ a principal driver of these individuals’ premature mortality.^9^

## Evidence of the diabetes risk of antipsychotics

Although diabetes may develop soon after antipsychotic exposure,^10^ evidence from randomised controlled trials regarding the safety of antipsychotics mainly provides information about intermediate outcomes such as changes in blood glucose levels.^11^ Therefore, evidence implicating SGAs as a cause of diabetes is overwhelmingly based on observational studies,^4,7^ many involving cross-sectional analyses^12^ and all strongly relying on the correctness of strong parametric assumptions encoded in statistical models. In addition, a majority of studies have used pooled comparisons, e.g. any or specific SGAs compared with any first-generation drug,^13^ or compared with no antipsychotic;^14^ these studies have limited utility for selecting specific drugs or for those who cannot forgo antipsychotic treatment. Moreover, little evidence exists on the effects of exposure duration.

## Present work

The evidence on the diabetes risk associated with antipsychotics has implications for multiple constituencies, including patients and their families, clinicians, guideline developers, the FDA and other regulatory agencies, and payers designing drug coverage policies. Hence, continued efforts are needed to improve the extant SGA safety evidence. The availability of newer, more robust statistical approaches for causal inferences in observational studies provides an opportunity to provide better insights into the comparative risks of antipsychotics. Importantly, these approaches facilitate accurate accounting for the duration of exposure. Here, using robust causal estimation methods, we examined the association between continuous monotherapy with aripiprazole or olanzapine and diabetes in a racially and ethnically diverse, publicly insured adult cohort with SMI. We focused on aripiprazole and olanzapine because of their polar rankings in terms of diabetes risk according to the available observational evidence, coupled with their having FDA approval for the three SMI disorders and ranking among the most highly utilised SGAs in the USA.^15^

## Method

### Data sources, study cohort and design

In the USA, Medicaid is a joint federal and state health insurance programme that covers low-income adults and disabled individuals, whereas Medicare is a federal health insurance programme for elderly (older than 64 years) and disabled non-elderly adults with past employment. Although many individuals are covered by either programme, some (‘dual eligibles’) are covered by both. Our data sources were 2008–2013 administrative billing data from Medicaid and Medicare for seven states (California, Georgia, Iowa, Mississippi, Oklahoma, South Dakota and West Virginia); for dual eligibles, information from both Medicare and Medicaid files was linked (see Supplementary Method 1 available at https://doi.org/10.1192/bjo.2024.727 for details of data sources). We included Medicare and dual Medicaid–Medicare beneficiaries aged 18–64 years living in those states, who between 2008 and 2013 (a) had any of three SMI diagnoses (schizophrenia, bipolar I disorder, or severe MDD as a proxy for treatment-resistant MDD) (see Supplementary Method 2 for details of our case ascertainment strategy) and (b) were observed to fill prescriptions for aripiprazole or olanzapine (index drugs).

We constructed a retrospective cohort of individuals who were ‘relatively new users’ of either drug, i.e. (a) they had no fills for any antipsychotics other than the index drug in the 6-month period preceding the index fill (pre-period), and (b) if more than one index drug fill was observed, the fills’ sum of days supplied did not exceed 30 in a 90-day period. Eligibility for the final cohort required (a) continuous enrolment in the 6-month pre-period and the 6-month period following the index fill date, and (b) absence in the pre-period of diabetes conditions including type 2 diabetes and associated conditions, as well as secondary diabetes, other cardiometabolic morbidity (dyslipidaemia, hypertension and cardiovascular disorders) associated with diabetes risk, or polycystic ovarian syndrome, as its management might involve antidiabetic drugs (Supplementary Method 2). Beneficiaries were followed for an observation period of up to 24 months unless one of the censoring events was observed; these, in hierarchical order, included (a) death, (b) end of the study period, (c) turning 65 years old, (d) loss of insurance coverage, (e) index drug discontinuation, (f) addition of another antipsychotic and (7) switching antipsychotics.

The final cohort included beneficiaries who, starting on the index fill date, were exposed to continuous olanzapine or aripiprazole monotherapy for up to 24 months (see next section for definition). Individuals could contribute to the cohort only once. See Supplementary Figure 1 for a flowchart describing the construction of the study cohort.

### Variables

#### Exposure

Exposure, measured monthly, was continuous monotherapy with olanzapine or aripiprazole for up to 24 months, with continuous monotherapy defined as index drug fills in the current and all previous months and no fills for other antipsychotics during that time.

#### Outcome

The outcome was newly diagnosed type 2 diabetes ascertained with ICD-9 diagnosis codes for type 2 and secondary diabetes observed (a) as primary diagnosis in ≥1 in-patient discharge claims; or (b) as primary or secondary diagnosis in ≥2 out-patient claims during a 12-month period, or in one out-patient claim if an oral antidiabetic National Drug Code was also observed. Insulin drugs were excluded based on the assumption that insulin-only treatment indicated type 1 diabetes.

#### Independent variables

Independent variables included race and ethnicity (non-Latinx White, hereafter White; and non-White), age and sex, assessed in the index month. The other variables, most time-varying, included health status, defined by (a) other chronic medical conditions potentially associated with diabetes or having the potential to affect service use and thus likelihood of diagnosis (e.g. malignancies), (b) risk factors for cardiometabolic morbidity (e.g. obesity) and (c) psychiatric comorbidity (e.g. other affective disorders); service use (psychiatric, injury-related, and non-psychiatric out-patient and acute use); metabolic testing (lipid or glucose laboratory tests); exposure to drugs with cardiometabolic effects (antidiabetic drugs, anti-hypertensive drugs, and other drugs such as mood stabilisers and antidepressants drugs with potential cardiometabolic effects); pre-period exposure to the index drug (days on drug); and year of index fill. Beneficiary state was also included in the models. Standardised mean differences (SMDs)^16^ for each independent variable between beneficiaries treated with aripiprazole versus olanzapine were computed. Values less than −0.10 and greater than 0.10 suggest unbalance between the two groups. See Supplementary Methods 2 and 3 for additional details.

### Statistical analysis

We estimated the index drugs’ diabetes risk for each month beginning in month 2 up to month 24. To ensure the outcome was temporally later than the exposure, continuous exposure of *n* months included the month of the index fill and the *n* − 1 months following. The *n*-month outcome was then measured in the following month. We estimated drug-specific risk for each study month, the restricted mean survival time (RMST), and 95% CIs for all quantities. The RMST measures the average number of diabetes-free months over the entire 24-month period. To examine how drug effects evolved over the study period, we also computed risk differences quantifying the absolute risk difference between olanzapine and aripiprazole in percentage points at a given monotherapy month, as well as risk ratios quantifying the relative difference between the drugs. For instance, if the 6-month diabetes risks were 4% and 2% for olanzapine and aripiprazole, respectively, the risk difference would indicate that olanzapine's risk was two percentage points higher than aripiprazole's, whereas the risk ratio would indicate that its risk was twice aripiprazole's risk. For rare outcomes, small risk differences can still correspond to large risk ratios. For all estimates, we additionally report 95% CIs.

Risk was estimated using targeted minimum loss-based estimation^17^ (TMLE) and adjusted for pre-treatment confounding as well as time-varying loss to follow-up. Unlike previously used approaches such as Cox proportional hazards that assume a parametric model, this approach makes minimal assumptions about the data-generation process. TMLE combines various estimates, each of which is derived flexibly using a combination of machine learning algorithms; these include the probability of remaining on treatment at a given study month, accounting for all previous information, and the probability of experiencing the outcome in a study month given treatment history and other covariates. The algorithms included random forests, neural networks, classification and regression trees, linear regression, logistic regression and a simple mean estimator; the Super Learner^18^ was used to find the best-fitting combination of these algorithms using two-fold cross-validation. Each of the individual algorithms used default tuning parameters, as implemented in the SuperLearner package (see Supplementary Method 4 for additional details).

#### Stratification

Analyses were stratified by primary diagnosis (schizophrenia, bipolar I disorder, severe MDD) to assess potential effect differences across these patient groups.

#### Sensitivity analyses

We conducted several analyses, each focusing on the impact of unmeasured confounding (see Supplementary Method 4 for additional details).

First, we estimated E-values, which are general tools for sensitivity analyses of unmeasured confounding that do not require assumptions about the nature of the unmeasured confounding.^19,20^ We used E-values to quantify how strong the association between an unmeasured confounder would have to be for both aripiprazole and diabetes (on the risk ratio scale) to explain the observed association. Large E-values suggest that considerable unobserved confounding would be required to change the findings.

Second, we examined adjusted influenza vaccination rates during the period of continuous monotherapy with each drug, using the same estimation technique as for the main analysis. As aripiprazole or olanzapine use should not affect the probability of influenza vaccination, observed differences may signal unmeasured differences between the two groups in frequency of healthcare system contacts.

Third, we examined rates of metabolic testing during the period of continuous monotherapy with each drug, estimated as the proportion of months with metabolic testing during the observation period. This analysis aimed to determine whether clinicians might have possessed different amounts of information suggesting potential diabetes risk for the two drug groups.

Last, we re-estimated all models among individuals aged 18–45 years with no previous index drug exposure: (a) this subgroup had a shorter life interval preceding their inclusion in our cohort, during which they might have developed diabetes risk factors, unobserved by us; and (b) clinicians may have had less information on risk signals, similarly unobserved by us.

### Ethics and consent statement

This study was reviewed and approved by the authors’ institutional review boards (RAND Human Subjects Protections Committee, Protocol#: 2015–0657, and Harvard University Faculty of Medicine Institutional Review Board, Protocol #: IRB16-0395). No informed consent is required for studies using anonymised previously collected data. This paper is an honest, accurate, and transparent account of the study being reported; no important aspects of the study have been omitted; and any discrepancies from the study as planned have been explained.

## Results

### Characteristics of the study cohort

Our study cohort included 21 293 beneficiaries receiving aripiprazole (54%) or olanzapine (46%), of whom 67% were White, half were female and 52% were older than 45 years. More than half of the cohort (52%) had a schizophrenia diagnosis, and 19% and 22% had a psychiatric or chronic medical comorbidity, respectively. Pre-period index drug exposure was observed in 76% of the cohort, and only 18% had previously undergone metabolic testing (Table 1). The diagnostic groups differed, with the largest differences observed between the schizophrenia group and the other two groups. For example, individuals in the schizophrenia group were more likely to be non-White and had lower rates of comorbidity and greater pre-period index drug exposure (Table 1). With respect to SMDs, the aripiprazole and olanzapine groups were relatively balanced. Some of the largest unbalance was observed for sex distribution, with more females in the aripiprazole group, and for days of pre-period index antipsychotic drug exposure, with the olanzapine group having more exposure, particularly in the severe MDD group.

**Table 1:** Demographic, clinical, and healthcare characteristics of the study cohort by primary diagnosis and drug type.

	Bipolar I disorder (*N* = 5500)					Severe MDD (*N* = 4707)					Schizophrenia (*N* = 11 086)						
Characteristic	Aripiprazole (*N* = 3582)		Olanzapine (*N* = 1918)			Aripiprazole (*N* = 3489)		Olanzapine (*N* = 1218)			Aripiprazole (*N* = 4384)		Olanzapine (*N* = 6702)			All (*N* = 21 293)	
	*N*	%	*N*	%	SMD^a^	*N*	%	*N*	%	SMD^a^	*N*	%	*N*	%	SMD^a^	*N*	%
Age, years																	
18–30	444	12.4	202	10.5	−0.058	244	7.0	84	6.9	−0.004	687	15.7	780	11.6	−0.113	2441	11.5
31–45	1506	42.0	704	36.7	−0.107	1065	30.5	351	28.8	−0.037	1812	41.3	2394	35.7	−0.114	7832	36.8
>45	1632	45.6	1012	52.8	0.145	2180	62.5	783	64.3	0.037	1885	43.0	3528	52.6	0.194	11 020	51.8
Female	2498	69.7	939	49.0	−0.450	2455	70.4	620	50.9	−0.427	1914	43.7	1921	28.7	−0.302	10 347	48.6
Race and ethnicity																	
Non-White	812	22.7	474	24.8	0.049	903	25.9	403	33.1	0.165	1759	40.1	2714	40.6	0.009	7065	33.2
White	2763	77.3	1441	75.2	−0.050	2583	74.1	813	66.9	−0.164	2624	59.9	3974	59.4	−0.010	14 198	66.8
Health status																	
Other chronic medical conditions	917	25.6	470	24.5	−0.025	1040	29.8	341	28.0	−0.039	790	18.0	1043	15.6	−0.062	4601	21.6
Risk factors for cardiometabolic morbidity	111	3.1	43	2.2	−0.052	127	3.6	14	1.1	−0.134	95	2.2	77	1.1	−0.075	467	2.2
Psychiatric comorbidity	709	19.8	372	19.4	−0.010	973	27.9	298	24.5	−0.076	766	17.5	936	14.0	−0.092	4054	19.0
Metabolic testing	748	20.9	401	20.9	0.000	707	20.3	243	20.0	−0.007	741	16.9	951	14.2	−0.072	3791	17.8
Payer																	
Dual	2436	68.0	1308	68.2	0.004	2328	66.7	867	71.2	0.095	3519	80.3	5250	78.3	−0.050	15 708	73.8
Medicare	1146	32.0	610	31.8	−0.004	1161	33.3	351	28.8	−0.095	865	19.7	1452	21.7	0.050	5585	26.2
Index (fiscal) year																	
2009	1061	29.6	671	35.0	0.118	745	21.4	427	35.1	0.334	1741	39.7	3023	45.1	0.110	7668	36.0
2010	782	21.8	371	19.3	−0.061	694	19.9	216	17.7	−0.055	928	21.2	1138	17.0	−0.103	4129	19.4
2011	587	16.4	301	15.7	−0.019	640	18.3	192	15.8	−0.065	619	14.1	886	13.2	−0.026	3225	15.1
2012	518	14.5	227	11.8	−0.077	591	16.9	151	12.4	−0.120	508	11.6	709	10.6	−0.031	2704	12.7
2013	471	13.1	235	12.3	−0.024	561	16.1	170	14.0	−0.057	417	9.5	623	9.3	−0.007	2477	11.6
2014	163	4.6	113	5.9	0.062	258	7.4	62	5.1	−0.088	171	3.9	323	4.8	0.046	1090	5.1
Drugs with cardiometabolic effects, any exposure																	
Antidiabetic drugs	176	4.9	60	3.1	−0.083	218	6.2	53	4.4	−0.075	219	5.0	214	3.2	−0.083	940	4.4
Other drugs for cardiometabolic disorders	914	25.5	475	24.8	−0.016	1230	35.3	374	30.7	−0.096	968	22.1	1439	21.5	−0.014	5400	25.4
Other drugs with cardiometabolic effects	2806	78.3	1325	69.1	−0.223	2962	84.9	952	78.2	−0.187	2622	59.8	3424	51.1	−0.177	14 091	66.2
Index antipsychotic drug exposure (pre-period)																	
Any exposure	2466	68.8	1451	75.7	0.149	1956	56.1	897	73.6	0.353	3596	82.0	5768	86.1	0.107	16 134	75.8
	Mean	s.d.	Mean	s.d.	SMD^a^	Mean	s.d.	Mean	s.d.	SMD^a^	Mean	s.d.	Mean	s.d.	SMD^a^	Mean	s.d.
Days	83.04	71.10	101.76	72.95	0.26	65.28	71.01	97.99	73.74	0.46	107.32	68.26	123.14	66.38	0.23	100.29	72.37
Service use																	
Psychiatric in-patient days	0.043	0.645	0.057	0.742	0.022	0.068	0.944	0.252	2.906	0.195	0.108	1.538	0.145	1.829	0.024	0.106	1.514
Injury-related in-patient days	0.050	0.605	0.099	1.891	0.081	0.066	0.812	0.172	1.782	0.131	0.057	0.944	0.078	1.201	0.022	0.074	1.145
Non-psychiatric in-patient days	0.496	3.074	0.576	3.644	0.026	0.628	3.333	0.914	4.922	0.086	0.449	3.196	0.505	4.093	0.018	0.542	3.657
Psychiatric emergency department visits	0.073	0.325	0.099	0.440	0.080	0.073	0.372	0.078	0.334	0.013	0.083	0.378	0.075	0.374	−0.021	0.078	0.371
Injury-related emergency department visits	0.080	0.406	0.083	0.387	0.007	0.057	0.270	0.053	0.252	−0.015	0.056	0.273	0.038	0.232	−0.066	0.057	0.299
Non-psychiatric emergency department visits	0.515	1.372	0.519	1.454	0.003	0.449	1.257	0.424	1.225	−0.020	0.382	1.116	0.271	0.959	−0.099	0.395	1.186
Psychiatric out-patient visits	0.546	2.863	0.530	2.619	−0.006	1.154	4.327	0.949	4.516	−0.047	0.642	4.170	0.575	4.527	−0.016	0.696	4.038
Injury-related out-patient visits	0.086	0.637	0.056	0.416	−0.047	0.072	0.416	0.062	0.371	−0.024	0.050	0.345	0.038	0.335	−0.035	0.057	0.425
Non-psychiatric out-patient visits	3.321	4.131	2.646	3.688	−0.163	3.954	4.500	3.279	4.206	−0.150	2.125	4.057	1.556	3.902	−0.140	2.560	4.172

MDD, major depressive disorder; SMD, standardised mean difference.

a. SMD represents the mean in the olanzapine group minus the mean in the aripiprazole group, standardised by the standard deviation in the aripiprazole group.

### Risk of type 2 diabetes

#### Schizophrenia group

**Fig. 1:**
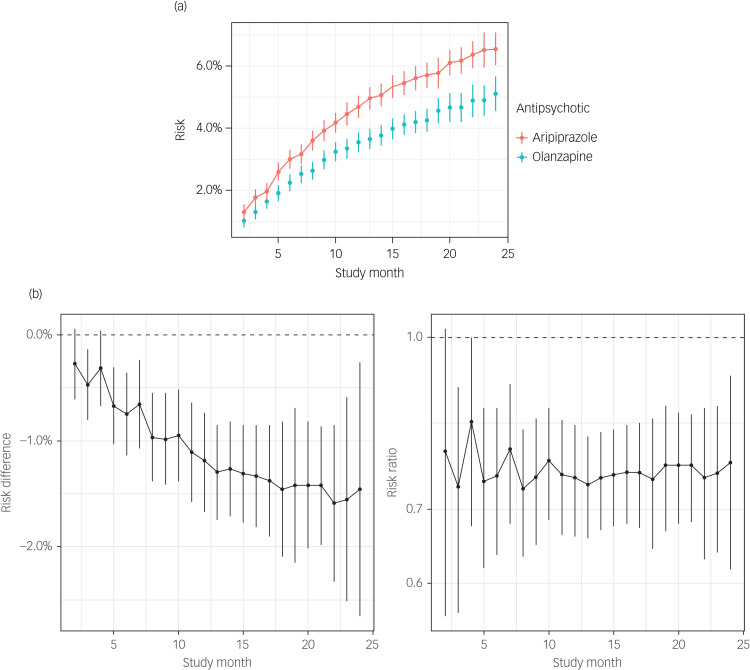
(a) Risk estimates and 95% CI for diabetes risk by study month in individuals with schizophrenia treated continuously with aripiprazole (red) or olanzapine (blue). Vertical line ranges correspond to 95% pointwise CI. (b) Differences in (left panel) and ratios of (right panel) diabetes risk by study month in individuals with schizophrenia treated continuously with olanzapine compared with the risk of treatment with aripiprazole (reference group).

The average number of diabetes-free months during the 24-month observation period was estimated to be 22.2 (95% CI [22.1, 22.3]) if all patients received olanzapine monotherapy, compared with 22.0 [21.9, 22.0] if all received aripiprazole monotherapy (Fig. 1(a)), yielding a RMST difference of 0.25 months [0.14, 0.36]. By month 24, the estimated olanzapine diabetes risk (5.1%) was 1.5 percentage points lower [−2.7, −0.3] and about 0.77 [0.62, 0.92] times that of aripiprazole (Fig. 1(b)). The increased risk with aripiprazole was observed from the start of the observation period and remained high across the study period (Fig. 1), with the largest risk difference at the end of the 24-month period.

#### Bipolar I disorder group

**Fig. 2:**
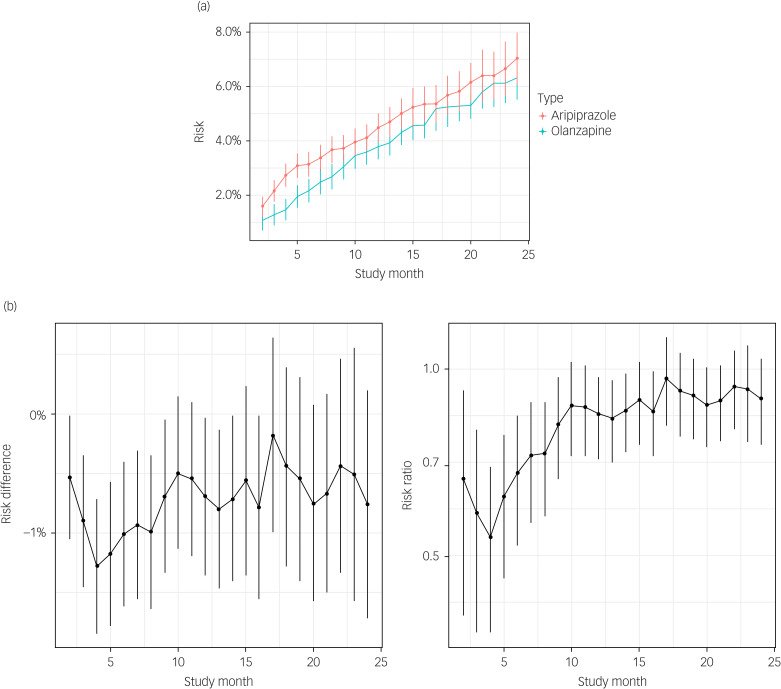
(a) Risk estimates and 95% CI for diabetes risk by study month in individuals with bipolar I disorder treated continuously with aripiprazole (red) or olanzapine (blue). Vertical line ranges correspond to 95% pointwise CI. (b) Differences in (left panel) and ratios of (right panel) diabetes risk by study month among individuals with bipolar I disorder treated continuously with olanzapine compared with the risk of treatment with aripiprazole (reference group)

Although similar findings were observed for patients with bipolar I disorder, the 24-month RMST [95% CI] estimates for olanzapine versus aripiprazole monotherapy were more variable (22.1 [22.0, 22.2] *v.* 21.9 [21.8, 22.1]; difference: 0.16 months [0.02, 0.31]) (Fig. 2(a)). By month 24, the estimated olanzapine diabetes risk (6.3%) was about 0.8 percentage points lower [−1.7, 0.2] and about 0.90 times [0.76, 1.04] that of aripiprazole (Fig. 2(b)). The increased risk with aripiprazole was observed at all points of the study period (Fig. 2), but the differences were largest at the beginning.

#### Severe MDD group

**Fig. 3:**
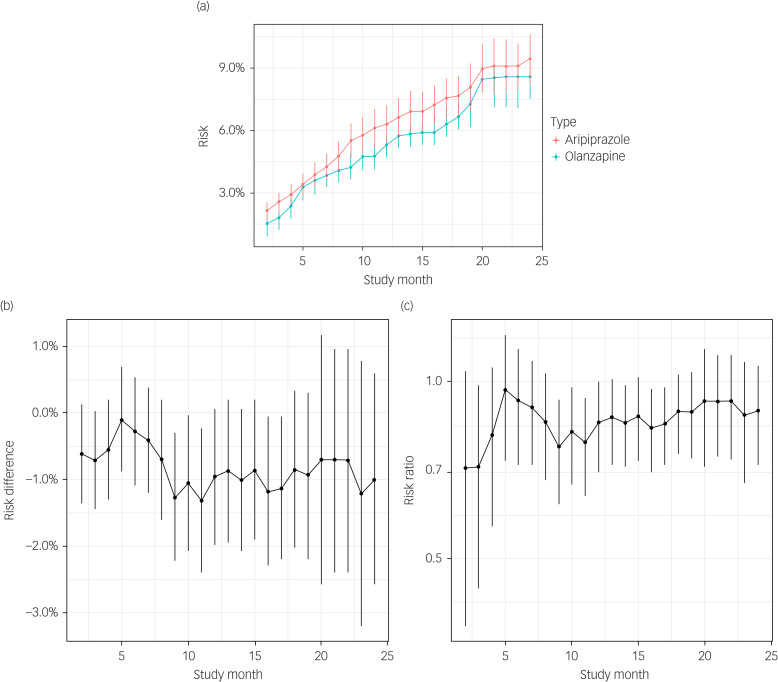
(a) Risk estimates and 95% CI of diabetes risk by study month among individuals with severe MDD treated continuously with aripiprazole (red) or olanzapine (blue). Vertical line ranges correspond to 95% pointwise CI. (b) Differences in (left panel) and ratios of (right panel) diabetes risk by study month among individuals with severe MDD treated continuously with olanzapine compared with the risk of treatment with aripiprazole (reference group)

The pattern of findings was similar among patients with severe MDD. The 24-month RMST [95% CI] estimates for olanzapine versus aripiprazole monotherapy were 21.8 [21.6, 21.9] *v.* 21.6 [21.4, 21.7], for a difference of 0.22 months [0.01, 0.44] (Fig. 3(a)). By month 24, the estimated olanzapine diabetes risk (8.6%) was about one percentage point lower [−2.6, 0.6] and about 0.89 times [0.72, 1.06] that of aripiprazole (Fig. 3(b)).

### Sensitivity analyses

The E-value analysis of unmeasured confounding showed that most E-values were above 1.5 and some were greater than 2.0 for all three diagnosis groups (Supplementary Figure 2). One E-value was greater than 3.0 for the risk ratio estimated in the bipolar group. This suggests that after accounting for all observed confounders, unobserved confounding would need to be at least moderately strong – and, in some cases, quite strong – to explain the observed associations. To contextualise the strength of this association, consider the marginal strength of association with the observed confounders listed in Table 1. Only three of the binary characteristics had a marginal association as strong as 1.5 with aripiprazole, and only one (risk factors for cardiometabolic morbidity, 1.4% among olanzapine patients and 2.9% among aripiprazole patients) had an association as strong as 2.0.

Olanzapine-treated patients received influenza vaccination sooner than aripiprazole-treated patients. Relative to aripiprazole-treated patients, the number of olanzapine-treated vaccination-free months was estimated to be 0.28 months lower [−0.40, −0.17] in the schizophrenia group (Supplementary Figure 3(a)); 0.23 months lower [−0.47, 0.01] in the severe MDD group (Supplementary Figure 3(b)); and essentially no different in the bipolar I disorder group (RMST difference = −0.05 months [−0.28, 0.18]) (Supplementary Figure 3(c)).

Olanzapine-treated patients did not receive more metabolic testing compared with aripiprazole-treated patients. The average rates of metabolic testing were 6.4% for aripiprazole-treated patients and 5.3% for olanzapine-treated patients in the schizophrenia group, with rates closer in those with bipolar I disorder (aripiprazole 7.9% *v.* olanzapine 8.0%) and severe MDD (aripiprazole 8.2% *v.* olanzapine 7.9%).

Among individuals aged 18–45 years with no previous index drug exposure, there was no strong evidence of diabetes risk differences between olanzapine and aripiprazole. The estimated RMST differences were −0.05 [−0.37, 0.26] for schizophrenia (Supplementary Figure 4(a)), −0.20 [−0.65, 0.25] for bipolar I disorder (Supplementary Figure 4(b)) and −0.00 [−0.31, 0.31] for severe MDD (Supplementary Figure 4(c)), with notably larger confidence intervals owing to the much smaller sample size.

## Discussion

In this study of publicly insured adults with schizophrenia, bipolar I disorder or severe MDD receiving continuous aripiprazole or olanzapine monotherapy over 24 months, we found a slightly higher diabetes risk associated with aripiprazole compared with olanzapine treatment regardless of diagnosis. This finding summarises the risk over the entire 24-month period, not just at 24 months. For instance, even if the two index drugs had similar risks of incident diabetes at 24 months, a lower RMST for aripiprazole would indicate that the risk occurred earlier in the study period in that group.

Evidence suggesting that olanzapine is more effective than aripiprazole for patients with schizophrenia^21,22^ elevates the significance of our findings, given the high rate of antipsychotic treatment failure in this population. In addition, the effectiveness advantage of olanzapine may have broader safety benefits, as suggested by evidence that clozapine, the most effective yet cardiometabolically riskiest antipsychotic, outperforms all others in terms of cardiometabolic drug adherence^23^ and with respect to all-cause and cardiovascular mortality.^24^

Our finding of a small associational advantage for olanzapine stands in contrast with current notions of comparative diabetes risk between the drugs.^4^ However, our study is not the first to challenge these notions. Other studies, both randomised controlled trials and observational studies,^12,25–27^ have found a lower-than-expected diabetogenic potential of olanzapine or a higher-than-expected risk for aripiprazole. In addition, despite robust evidence that olanzapine causes more weight gain than other antipsychotics, including aripiprazole,^28^ the relative contribution of weight-mediated effects to incident diabetes among antipsychotic-treated patients has not been fully elucidated. Although we cannot rule out that our finding may reflect selection that our robust methods were unable to address (see ‘Limitations’), if selection were driving this result, it would suggest that clinicians are highly skilled at matching patients to antipsychotics based on patients’ diabetes risk potential.

Few studies have evaluated the impact of antipsychotic exposure intensity (dose and/or duration) on diabetes risk among individuals with SMI, with most having focused on class or drug-specific dose effects (e.g. ref. ^29^). We are aware of only one duration-focused study, which found no association between exposure duration and diabetes risk in patients with schizophrenia.^30^ Our finding that for individuals with schizophrenia, the differences between the drugs were largest at the end of the 24-month period may reflect the duration dependence of the effects. That the opposite appeared to be true for individuals with bipolar I disorder or severe MDD might have been due to differences among the diagnostic cohorts; however, we did not have power to test this possibility.

We cannot contextualise our finding that the comparative diabetes risk between the drugs was relatively consistent across the three disorders, as we are unaware of similar analyses in the extant evidence. Although variable cardiometabolic risk by SMI diagnosis is plausible,^7^ the available evidence suggests similar antipsychotic-related diabetes risk across SMI diagnoses.^12^ However, the evidence on the diabetes risk of antipsychotics in individuals with major affective disorders is relatively small and not entirely consistent with that produced by schizophrenia studies in terms of specific drugs’ risks.^31^

### Limitations

Our study had some limitations. First, as in all observational studies, we were unable to rule out unobserved confounding, that is, confounding due to risk factors such as family history that were unobserved to us owing to incomplete clinical information but known to clinicians, or caused by misclassification of measured confounders. However, the E-value analysis revealed that unobserved confounding would need to be at least moderately strong to explain the observed associations, suggesting that the risk of unobserved confounding was not severe. In other words, even stronger unobserved confounding would be required if the risk associated with olanzapine was actually much higher than for aripiprazole. Other sensitivity analysis results yielded small differences in diabetes risk between aripiprazole and olanzapine in the subgroup at lowest risk for confounding by indication (younger patients with no previous index drug exposure); however, the small sample size precluded a definitive finding. In addition, although we did find some differences between the two drug groups in receipt of influenza vaccination, suggesting that there may have been unobserved differences between the drug groups, these findings also suggest that the higher diabetes risk of aripiprazole is not driven by these individuals having more healthcare system contacts and thus more chance of being diagnosed with diabetes. We note that our approach differed from those of prior observational studies that made restrictive parametric assumptions and did not characterise the effects of antipsychotic exposure duration, owing to conflation of long-term users with those who switched or added antipsychotics. Our approach made very few assumptions about the relationships among patient characteristics, antipsychotic exposure and diabetes risk, and we characterised diabetes risk specifically for continuous monotherapy measured over time. Second, we could not rule out the possibility of informative censoring, where clinicians proactively transition patients who are likely to be diagnosed with diabetes off the index drug. This informative censoring would only be concerning if (a) it was more likely to happen in the olanzapine-treated group than in the aripiprazole-treated group and (b) the diabetes risk that caused the clinician to discontinue the index drug was not captured in the time-varying information available in our database (which includes risk factors for cardiometabolic morbidity and metabolic testing longitudinally), neither of which we could rule out. Third, we only assessed duration and not dose when evaluating the effects of exposure intensity on diabetes risk.

### Clinical implications

Using robust methods and accounting for exposure duration effects, our study found a slightly higher risk of type 2 diabetes associated with aripiprazole compared with olanzapine monotherapy regardless of diagnosis, suggesting that, at best, olanzapine may have a diabetes risk comparable with that of aripiprazole. If this result was due to unmeasured selection, it would suggest clinician success in identifying good olanzapine candidates. Confirmatory research is needed, and clinicians should continue to regard olanzapine as a second-line antipsychotic for individuals at higher risk of treatment-emergent diabetes. However, this insight suggests a potentially larger role for olanzapine in the treatment of well-selected patients, particularly for those with schizophrenia, given the drug's effectiveness advantage among them. Ultimately, prescribing decisions should be guided by the best evidence on the risks and benefits associated with each drug, given each patient's risk profile, diagnosis and illness presentation.

## Supporting information

Agniel et al. supplementary material Agniel et al. supplementary material

## Data Availability

No data used in the study, including individual participant data, can be made available to others, because our data use agreement with the data purveyor (US Center for Medicare and Medicaid Studies) prohibits data sharing. Readers can visit https://www.nia.nih.gov/research/dbsr/obtaining-cms-data-your-research to learn how to access the data and obtain a description of the data, including the data elements.
