# Supplementary material for: Revisiting diabetes risk of olanzapine versus aripiprazole in serious mental illness care
Source: BJPsych Open. 2024 Aug 8;10(5):e144. doi: 10.1192/bjo.2024.727 (PMC11698215; doi:10.1192/bjo.2024.727)
Supplement: Agniel et al. supplementary material [file S2056472424007270sup001.docx]

The manuscript was written in accordance with STROBE reporting guidelines – we report no deviation from these guidelines.

**Supplementary Method 1. Data Sources**

Our data sources were 2008-2013 Medicaid data (Medicaid Analytic eXtract data extracted from the states’ Medicaid Statistical Information System) and Medicare data for California, Georgia, Iowa, Mississippi, Oklahoma, South Dakota, and West Virginia. We selected these states because of their publicly insured populations’ racial and ethnic diversity. Both datasets contain information on eligibility, demographic characteristics, diagnoses, and service and pharmacy utilization. Medicaid and Medicare data for dual eligibles were linked through a common identifier. Analyses focused on fee-for-service (FFS) beneficiaries because managed care data were less complete than FFS claims data for the earlier years of our study period. Continuous enrollment was defined as enrolled in Medicare Parts A, B, and D.

**Supplementary Method 2. Ascertainment of Diagnoses for Inclusion, Exclusions, and Variable Construction**

We captured diagnostic information from claims indicating inpatient or outpatient encounters with medical professionals. If inpatient admissions were observed, we looked for diagnoses in claims found in the Medpar (Medicare) and Inpatient (MAX/Medicaid) files. For outpatient encounters, we looked for diagnoses in outpatient and professional claims found in the Outpatient and Carrier files (Medicare), and claims found in the Other Therapy file (MAX/Medicaid). Given that the MAX data include a maximum of two diagnoses per claim, we examined the first two Medicare diagnoses out of a maximum of 25 to balance the observable information among the payers.

We ascertained SMI diagnoses by requiring at least one primary inpatient discharge or two primary or secondary outpatient ICD-9 diagnostic codes in claims from two different days indicating *schizophrenia* (295.0−295.9), *bipolar I disorder* (296.0, 296.1, 296.4−296.7), or *severe MDD* (296.2x and 296.3x, with 5^th^ digit indicating severe subtype with or without psychosis) during the 12-month period surrounding the index aripiprazole or olanzapine fill. Beneficiaries lacking 5^th^ digit codes met criteria if MDD codes were observed as primary discharge diagnosis in ≥ 2 occasions, or if ≥ 1 claim with electroshock therapy, trans-cranial magnetic stimulation, or suicide-related injury codes were observed during the 12-month period. Although SGAs are FDA-approved only for antidepressant treatment augmentation, we did not require observation of a concurrent antidepressant because we might have missed antidepressant drugs prescribed prior to the six-months preceding the SGA fill and discontinued due to inefficacy or intolerance.

Beneficiaries qualifying for more than one of these diagnoses were assigned a single primary diagnosis based on phenomenological hierarchy (schizophrenia highest and severe MDD lowest).

The conditions used for exclusions (see Box 1^1^) included our outcomes and related cardiometabolic morbidity as well as conditions such as polycystic ovarian syndrome that may complicate our outcome assessment. Case ascertainment required observation of at least one claim with a qualifying ICD-9 diagnosis in any position during the 6-month pre-period.

**BOX 1. CONDITIONS USED FOR EXCLUSIONS**

| 1. Dyslipidemia  2. Hypertension (essential)  3. Diabetes conditions  Type 2 Diabetes  Secondary diabetes  Polyneuropathy in diabetes  Diabetic retinopathy  Diabetic cataract  Peripheral angiopathy, other  4. Coronary Artery Disease  Acute myocardial infarction  Other acute/subacute disease  Angina pectoris  Coronary atherosclerosis Other coronary artery disease  Other chronic disease  Old infarction & sequelae  5. Heart Failure  Heart failure  Heart failure with complications | 272.x [x=0,1,2,4]  401.x  250.x0, x2  249.x0, x1  357.2  362.0x  366.41  443.81  410.0x-410.9x  411.x [x=0,1]; 411.8x [x=1,9]  413.0; 413.9  414.0x  414.x [x=2-4]  414.8, 414.9  412, 429.79  428.xx  402.x1,404.x1, x3 | 6. Stroke & Other cerebrovascular disorder  Intracerebral hemorrhage  Precerebral artery disease  Cerebral artery disease  Transient ischemic accident  Other cerebrovascular disorder  7. Other Vascular  Hypertensive organ disease  Cardiovascular disease, other  Atherosclerosis  Peripheral vascular disease  Embolisms/thrombosis  8. Polycystic Ovarian Syndrome | 431  433.0x, 1x, 2x, 3x, 8x, 433.9x  434.0x-9x  435.x  436, 437.x [x=0,1,2,8], 438.xx  402.x0, 403.x0,  404.x0, x2  429.2  440.x [x=0,1,4,8,9],  440.2x, 440.3x  443.9  444.0x, 445.xx  256.4 |
| --- | --- | --- | --- |

**Supplementary Method 3. Independent Variables**

*Race and ethnicity (assessed in the index month),* described as non-Latinx Whites and non-White (non-Latinx Blacks, Latinx, and those of missing/other race and ethnicity). Greater disaggregation of the non-White category was infeasible due to insufficient sample size. We used the Research Triangle Institute (RTI) variable,^2^ one of the two race and ethnicity variables included in the Medicare data. The original variable captures self-reported race and ethnicity information collected by the Social Security Administration at the time of application for a Social Security Number and transferred to the Centers for Medicare & Medicaid Services’ Medicare program’s enrollment database. Because of incompleteness of this information, the Medicare program includes a second variable developed at the RTI that improves classification of Latinxs and Asians/Pacific Islanders through an imputation algorithm based on names from the US Census and geography. The RTI variable performs quite well relative to the gold standard (directly assessed self-reported race and ethnicity)^3^ and is the default variable used by the Centers for Medicare & Medicaid Services in its reports on racial and ethnic disparities among Medicare-covered individuals.^4^

*Other demographic (assessed in the index month),* including age (continuous) and sex.

*Health status (time-varying),* described through three binary yes/no composite variables composed of specific conditions whose ascertainment required observation of at least one claim with a qualifying diagnosis in any position during the 6-month pre-period (see Box 2^5, 6^): (a) other chronic medical conditions potentially associated with diabetes or with the potential to affect service utilization and thus likelihood of diagnosis; (b) risk factors for cardiometabolic morbidity; and (c) psychiatric comorbidity

*Service utilization (time-varying),* described through three count variables capturing psychiatric, injury-related, and non-psychiatric inpatient days, emergency department (ED) visits, and outpatient visits.^6^

*Metabolic testing (time-varying),* described through a binary yes/no variable that was met if ≥1 procedure codes for lipid or glucose laboratory tests were observed.

*Drugs with cardiometabolic effects, any exposure (time-varying),* described through three binary yes/no variables which captured observation of NDCs for (1) antidiabetic drugs, (2) anti-hypertensive drugs, and (3) other drugs with potential weight-related and cardiometabolic effects (see Box 3^5, 6^).

*Index antipsychotic drug exposure (assessed in the pre-period),* defined as days on the index (aripiprazole or olanzapine) drug.

*Time,* defined as the year of the index fill.

**BOX 2. HEALTH STATUS VARIABLES**

| **Other chronic medical conditions**  *1. Chronic Infections* | | *7. Autoimmune, connective tissue & related disorders* | |
| --- | --- | --- | --- |
| Tuberculosis  HIV  Viral hepatitis  *2. Endocrine disorders*  Goiter conditions  Thyrotoxicosis plus/minus goiter  Hypothyroidism  Thyroiditis  Other thyroid conditions  Juvenile Diabetes  Other pancreatic conditions  Other endocrine disorders  *3. Malignancies*    *4. Lung disorders*  COPD /Asthma  Pneumoconioses  Cystic fibrosis  Other lung disorders  *5. Gastro-Intestinal disorders*  Ulcers  Liver conditions  *6. Renal disorders*  Nephrotic syndrome  Chronic glomerulonephritis  Nephritis and nephropathy  Renal failure  Renal sclerosis  Disorders from renal  dysfunction | 010-018  042  070.2-070.9  240, 241  242  243, 244  245  246  250.x1, 250.x3  251; 577.1; 996.86, V42.83  226,227; 252-259  140.xx-208.xx, 230.xx-239.xx  490-496  500-505  277.0  506.4  531-534.9  570-573  581  582  583  585, 586  587  588 | Polyneuropathy (collagen disease)  Polyarteritis nodosa  Connective tissue disorders  Inflammatory polyarthropathies  Polymyalgia Rheumatica  Multiple sclerosis & related  *8. Neurological disorders*  Parkinson’s disease & related  Epilepsy  *9. Pregnancy-related*  Diabetes complications  Abnormal glucose tolerance  **Risk factors for cardiometabolic morbidity**  Obesity & Overweight  Metabolic syndrome  Pre-Diabetes & related  **Psychiatric comorbidity**  *1. Affective Disorders*  Neurotic depression  Cyclothymic disorder  Prolonged depressive reaction  Depressive disorder, other  *2. Other Psychoses*  Paranoid states  Other nonorganic psychoses  *3. Anxiety disorders*    *4. OCD*  *5. PTSD* | 357.1  446.0  710  714  725  340, 341  332.0, 332.1, 333  345  648.0x  648.8x  278.0x  277.7  790.2x  300.4  301.13  309.1  311  297  298  300.0x [x=0,1,2,9] 300.2x [x=0,1,2,3,9] 309.21  300.3x  309.81 |

**BOX 3. DRUGS WITH CARDIOMETABOLIC EFFECTS**

| **Drug Class** | **Drug Categories/Individual Drugs** |
| --- | --- |
| 1. Antidiabetic drugs | |
|  | Insulins |
|  | Thiazolidinediones |
|  | Sulfonylureas |
|  | Glucagon-like peptide-1 (GLP-1) receptor agonists |
|  | Sodium glucose co-transporter-2 (SGLT-2) inhibitors |
|  | Dipeptidyl peptidase-4 (DPP-4) inhibitors |
|  | Amylin analogues |
|  | Metiglinides |
|  | Alpha-glucosidase inhibitors |
|  | Biguanides (Metformin) |
| 1. Anti-hypertensive drugs | |
|  | Beta-Blockers |
|  | Alpha and Beta-Blockers |
|  | Thiazide Diuretics |
| 1. Other Drugs with potential cardiometabolic effects | |
| Obesity & weight-reducing drugs | Orlistat |
|  | Lorcaserin |
|  | Liraglutide (GLP-1 receptor agonist) |
|  | Phentermine (plus minus Topiramate) |
|  | Naltrexone/bupropion (ANTIDEPRESSANT DRUG) |
|  | Diethylpropion |
|  | Benzphetamine (STIMULANT) |
|  | Phendimetrazine |
| Appetite stimulants | Dronabinol |
|  | Megestrol |
| HIV drugs | Antiretrovirals (protease inhibitors) |
| Anti-inflammatory & Immunosuppressant drugs | Steroids |
| Hormonal drugs | Oral Contraceptives |
|  | Medroxyprogesterone |
| Neuro-muscular drugs | Cyclobenzaprine |
|  | Amantadine |
| Anticonvulsants | Valproic acid (MOOD STABILIZER) |
|  | Carbamazepine (MOOD STABILIZER) |
|  | Gabapentin |
|  | Pregabalin |
|  | Felbamate |
|  | Topiramate |
|  | Zonisamide |
| Mood Stabilizers | Lithium |
| Antidepressant drugs | Bupropion |
|  | Reboxetine |
|  | Mirtazapine |
|  | SRIs |
|  | Tricyclics |
|  | MAOIs |
| Stimulants | Amphetamine |
|  | Metamphetamine |
|  | Dextroamphetamine |
|  | Lisdexamfetamine |
|  | Methylphenidate |
|  | Dexmethylphenidate |
|  | Others |
| Miscellaneous | H2 Blockers, Antihistamines |

**Supplementary Method 4. Statistical analysis**

Risk was estimated using targeted minimum-loss based estimation (TMLE) via the lmtp package in R ^7^. We used Super Learner ^8^, an approach that finds an optimal combination of many machine learning algorithms, for this task. Super Learner combined linear regression, logistic regression, classification trees, neural nets, random forests, and a simple mean estimator. The tuning parameters for the classification trees were the defaults in the rpart package in R: complexity parameter = 0.01, the minimum number of observations required to split a node was 20, and the maximum tree depth was 30. The neural nets were feed-forward neural networks with a single hidden layer, as in the nnet package in R. Tuning parameters for the random forests (as in the ranger package in R) were: 500 trees, number of randomly sampled covariates was equal to the square root of the number of total predictors, and the minimum node size was 1. Standard errors for risk estimates, risk differences, and RMSTs were obtained from the standard deviation of the influence function for each estimator. Both TMLE and Super Learner use sample splitting to separate estimation of risk from nuisance function estimation. Two folds were used for sample splitting throughout. R version 4.2.0 was used for all analyses.

Due to the large number of functions estimated at many timepoints, we adopted two precautions to ensure the stability of our estimates. First, to guarantee that the risk at month m was not higher than at month m+1, we used isotonic regression ^9^, Second, because sample-splitting approaches can be sensitive to the particular split of the data, we estimated the risk at each month for each index drug five times for all analyses, using a different random split of the data for each. We then used the median of the five estimates and we used the corresponding method recommended in Chernozhukov et al. to combine the standard errors ^10^.

One of our sensitivity analyses involved comparing adjusted influenza vaccination rates between the two groups. The procedure and diagnosis codes used to define influenza vaccination were: CPT codes 90630, 90653-90660, 90662, 90672-90674, 90682, 90685-90689, 90694, 90756; HCPCS codes Q2034-Q2039 or G0008; ICD-9 code V05.9; and ICD-10 code Z23.

**Supplementary Figure 1. Cohort construction flowchart diagram.**

**192,579**

Number of beneficiaries with SMI who had 6 months of continuous enrollment in the pre-period, were enrolled during the index month, and were enrolled either for 6 months post index month or until death

**159,839**

Number of beneficiaries with a qualifying monotherapy episode

**98,230**

Number of beneficiaries with no pre-period conditions used for exclusions

**64,120**

Number of beneficiaries in Medicare or Dual in the index month

**21,293**

Number of beneficiaries who were on Olanzapine or Aripiprazole

**Supplementary Figure 2: E-value estimates of amount of unmeasured confounding that could explain the magnitude of observed risk ratio (solid black line) and unmeasured confounding that could explain the magnitude of upper limit of 95% confidence (dotted gray line)**

**Supplementary Figure 2a: E-value estimates for risk ratio among individuals with schizophrenia**

**
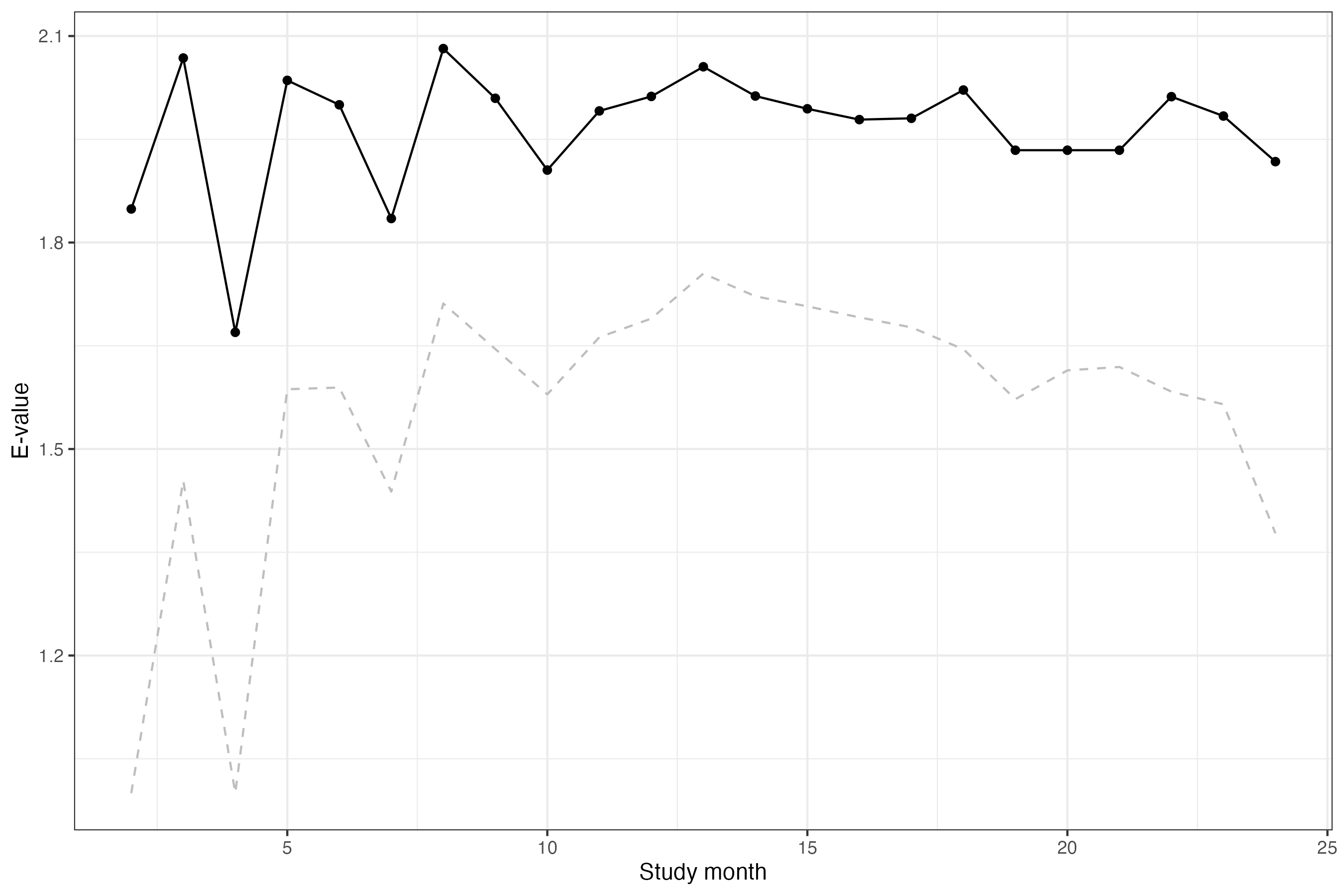
**

**Supplementary Figure 2b: E-value estimates for risk ratio among individuals with bipolar I disorder**

**
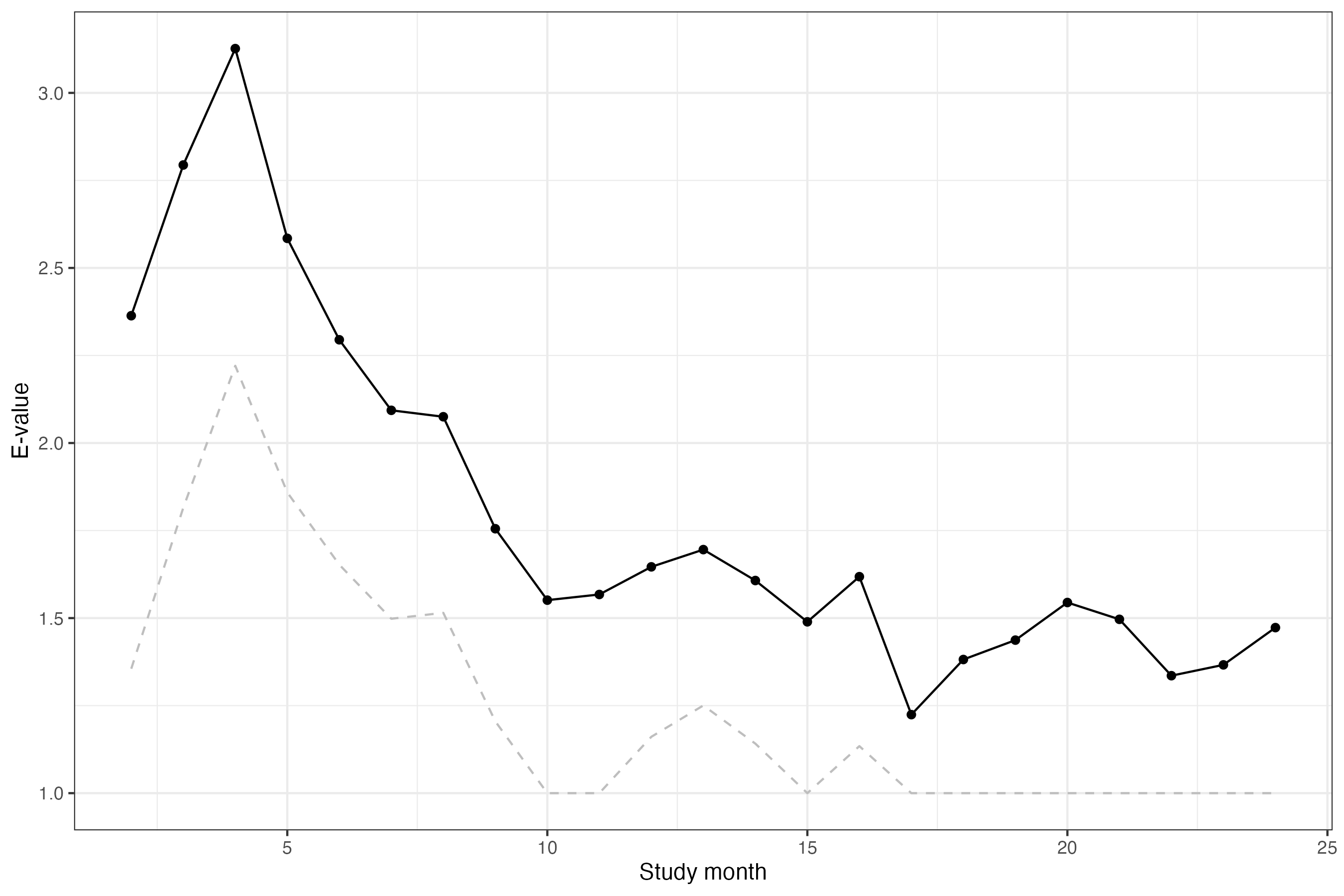
**

**Supplementary Figure 2c: E-value estimates for risk ratio among individuals with major depressive disorder**

**
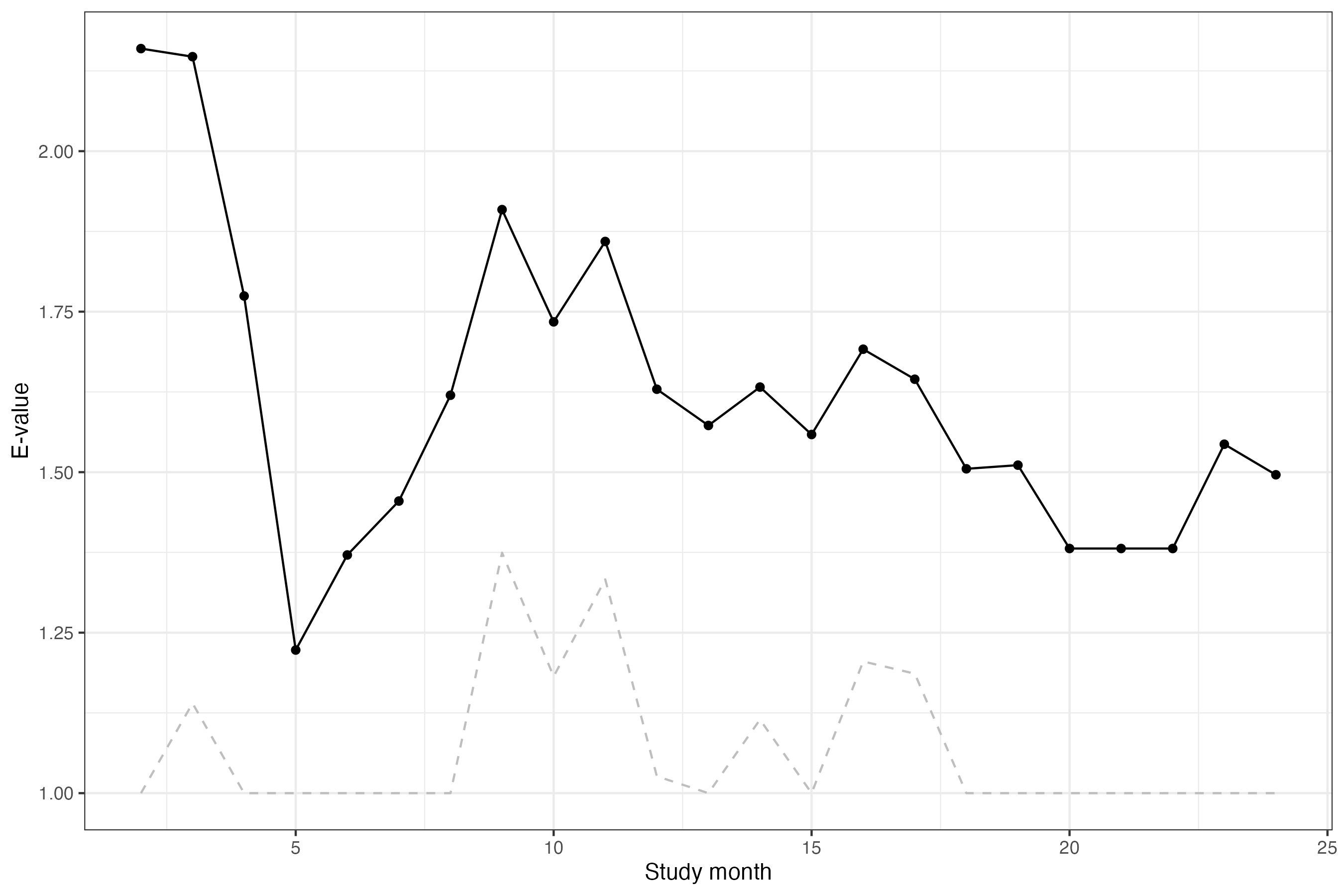
**

**Supplementary Figure 3. Estimates and 95% confidence intervals of standardized rates of influenza vaccination by study month when treating continuously with aripiprazole (red) or olanzapine (blue), by SMI diagnosis.**

**Supplementary Figure 3a: Standardized influenza rate estimates among individuals with schizophrenia.** Vertical line ranges correspond to 95% pointwise confidence intervals.

**
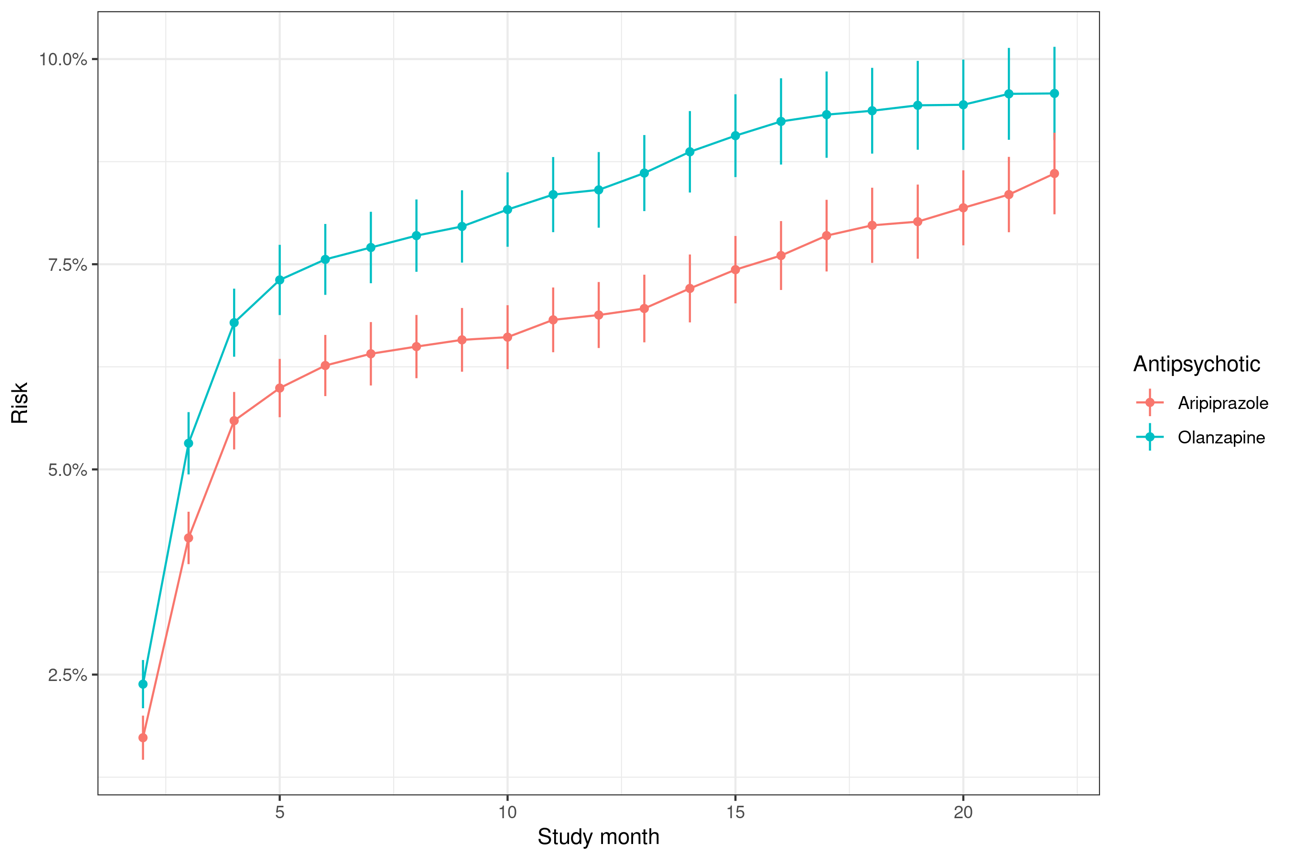
**

**Supplementary Figure 3b: Standardized influenza rate estimates among individuals with bipolar I disorder.** Vertical line ranges correspond to 95% pointwise confidence intervals.

**
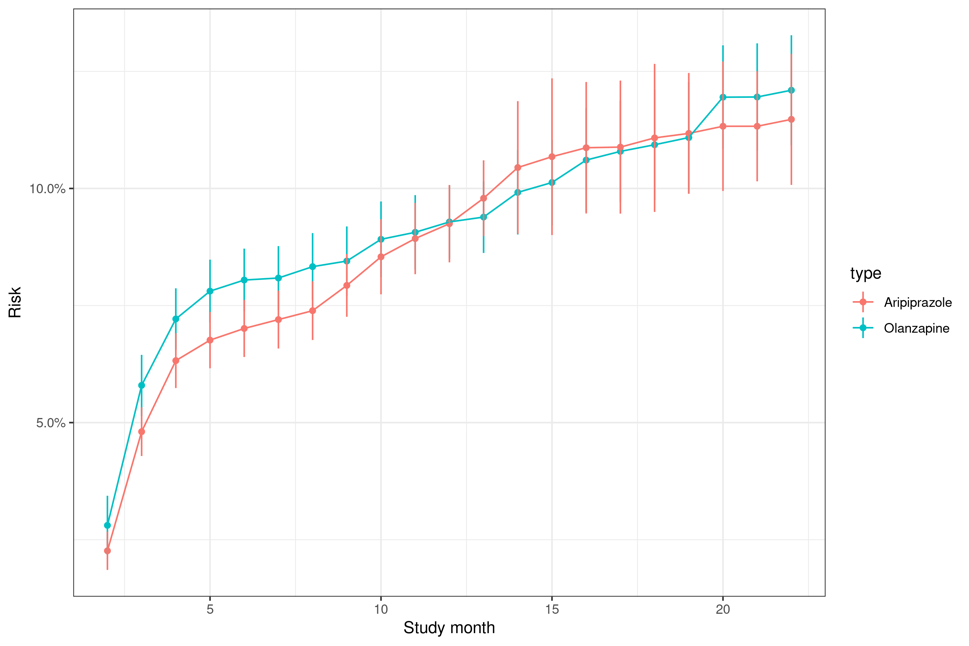
**

**Supplementary Figure 3c: Standardized influenza rate estimates among individuals with severe MDD.** Vertical line ranges correspond to 95% pointwise confidence intervals.

**
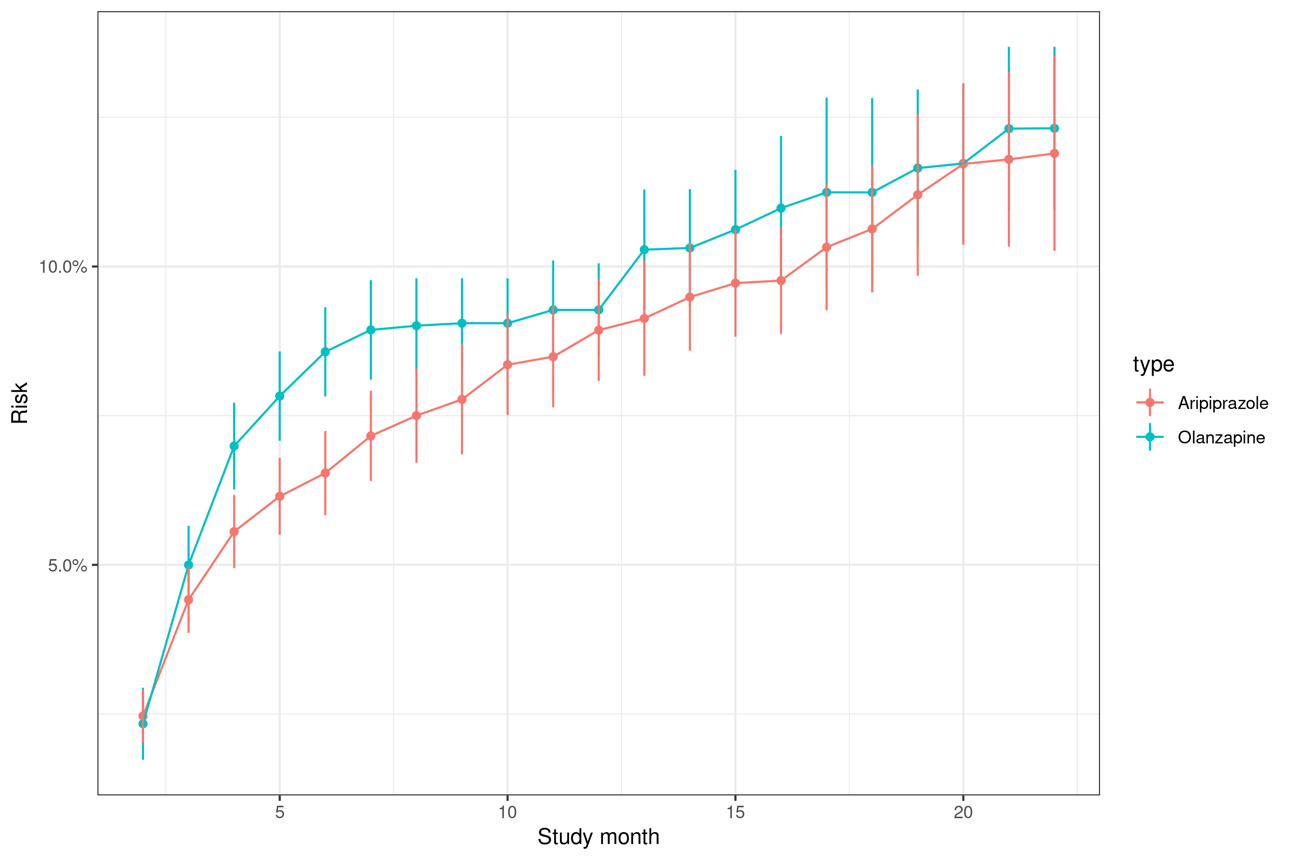
**

**Supplementary Figure 4. Estimates and 95% confidence intervals of diabetes risk by study month when treating continuously with aripiprazole (red) or olanzapine (blue), for the 18-45 age group with no previous exposure to the index drug, by SMI diagnosis.**

**Supplementary Figure 4a: Risk estimates among individuals with schizophrenia.** Vertical line ranges correspond to 95% pointwise confidence intervals.

**
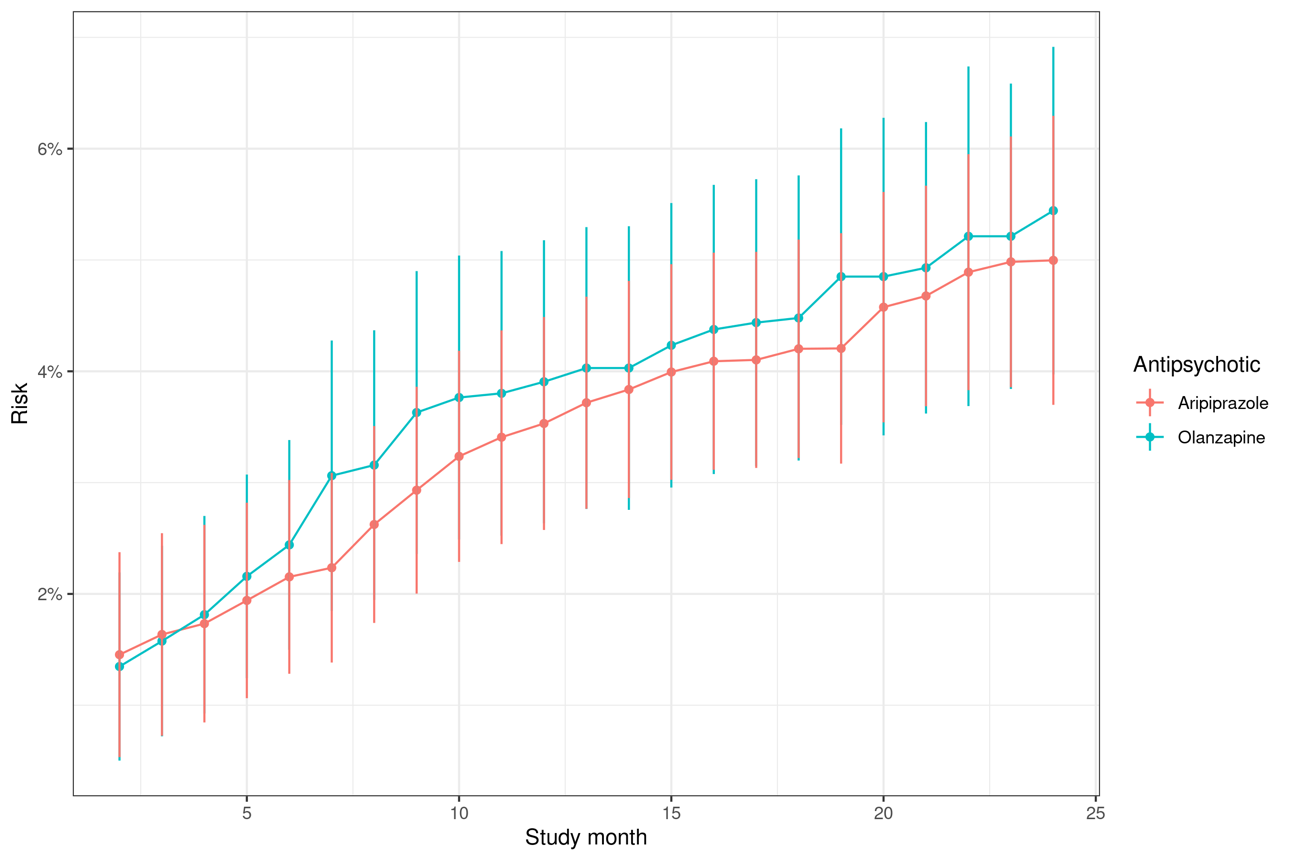
**

**Supplementary Figure 4b: Risk estimates among individuals with bipolar I disorder.** Vertical line ranges correspond to 95% pointwise confidence intervals.

**
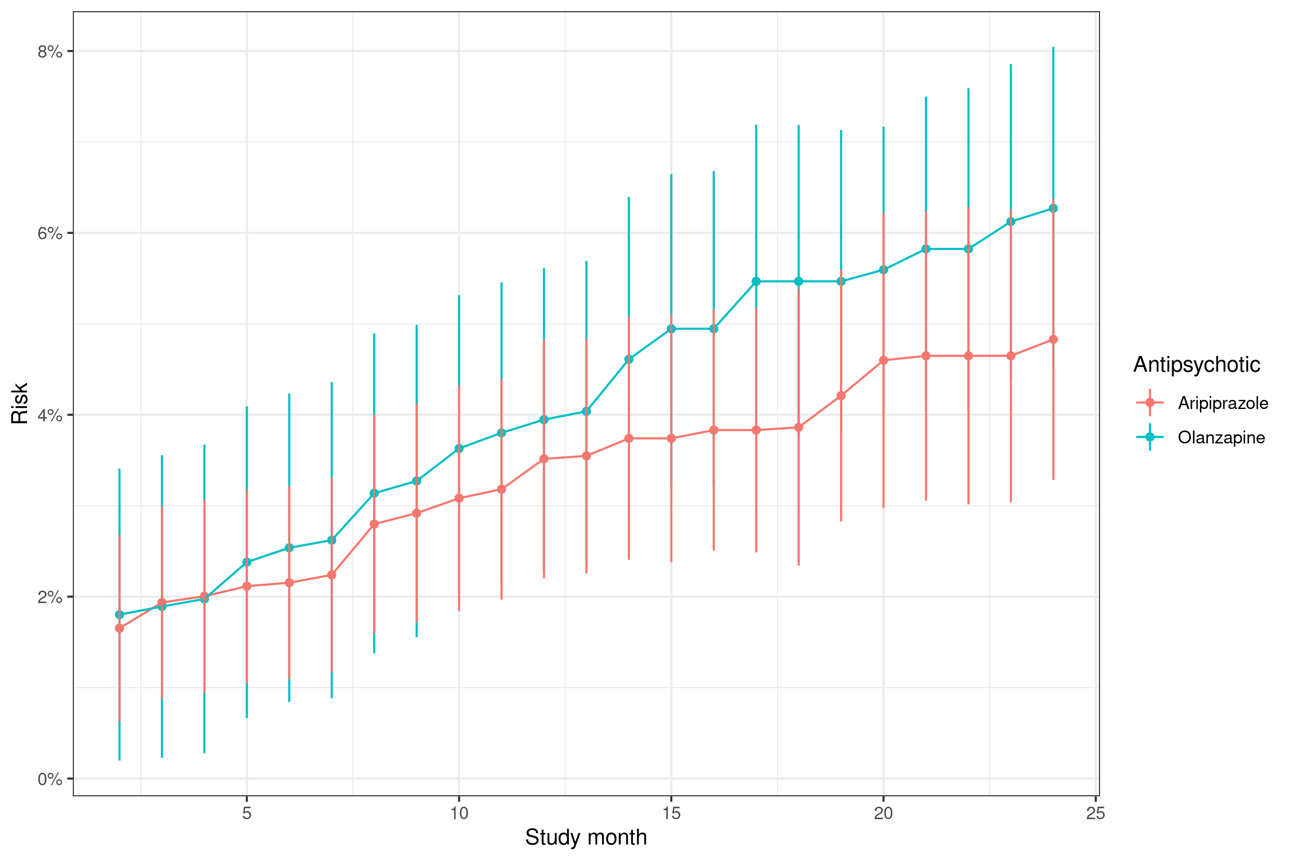
**

**Supplementary Figure 4c: Risk estimates among individuals with severe MDD.** Vertical line ranges correspond to 95% pointwise confidence intervals.

**
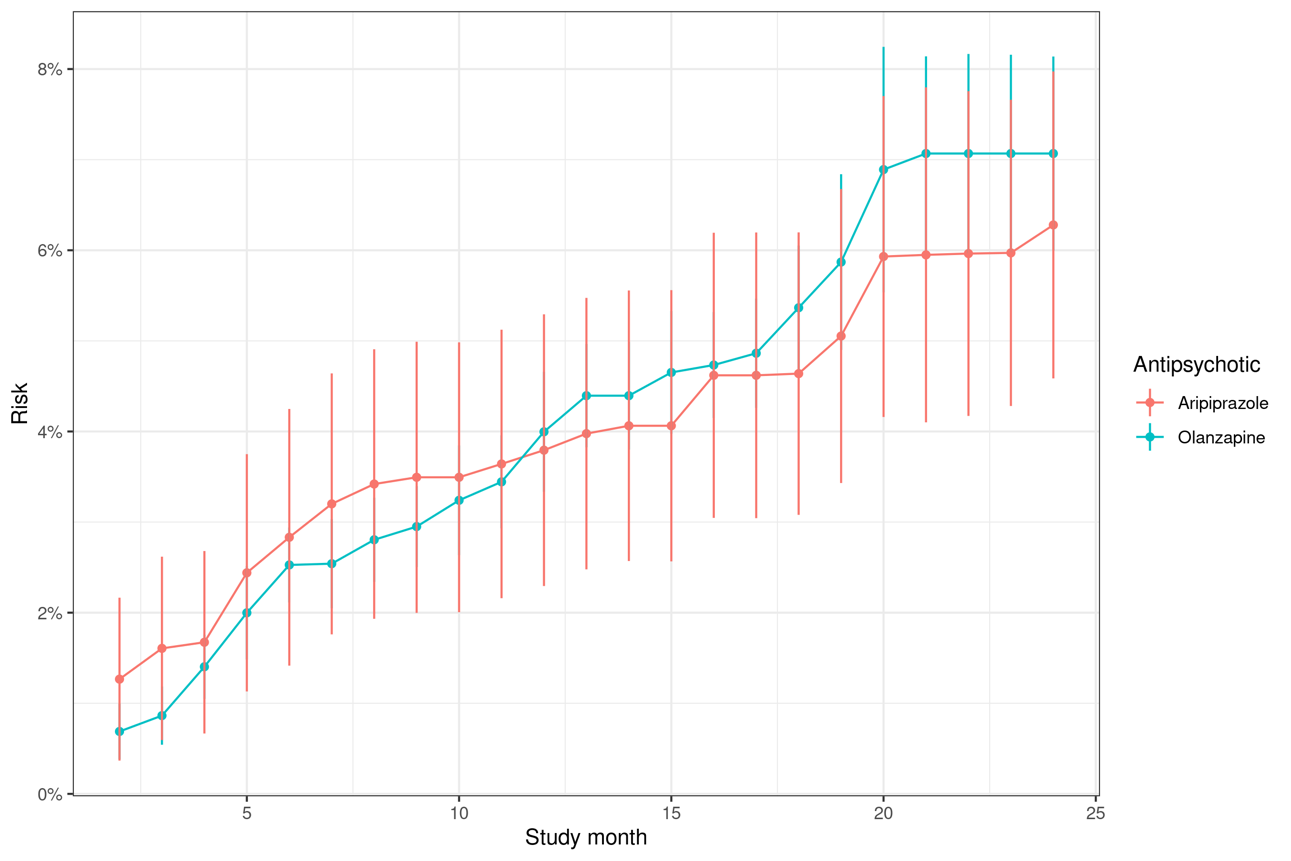
**

**Supplementary References**

1. Rubin DM, Kreider AR, Matone M, Huang Y-S, Feudtner C, Ross ME, et al. Risk for incident diabetes mellitus following initiation of second-generation antipsychotics among Medicaid-enrolled youths. *JAMA pediatrics* 2015; **169**: e150285-e.

2. Eicheldinger C, Bonito A. More accurate racial and ethnic codes for Medicare administrative data. *Health Care Financ Rev* 2008; **29**: 27-42.

3. Jarrín OF, Nyandege AN, Grafova IB, Dong X, Lin H. Validity of Race and Ethnicity Codes in Medicare Administrative Data Compared With Gold-standard Self-reported Race Collected During Routine Home Health Care Visits. *Medical Care* 2020; **58**: e1-e8.

4. Centers for Medicare & Medicaid Services Office of Minority Health. The Mapping Medicare Disparities Tool Technical Documentation, Version 8.0. July 30, 2020. . 2018.

5. Guo JJ, Keck PE, Jr., Corey-Lisle PK, Li H, Jiang D, Jang R, et al. Risk of diabetes mellitus associated with atypical antipsychotic use among Medicaid patients with bipolar disorder: a nested case-control study. *Pharmacotherapy* 2007; **27**: 27-35.

6. Bobo WV, Cooper WO, Stein CM, Olfson M, Graham D, Daugherty J, et al. Antipsychotics and the risk of type 2 diabetes mellitus in children and youth. *JAMA Psychiatry* 2013; **70**: 1067-75.

7. Díaz I, Williams N, Hoffman KL, Schenck EJ. Nonparametric causal effects based on longitudinal modified treatment policies. *Journal of the American Statistical Association* 2021: 1-16.

8. Van der Laan MJ, Polley EC, Hubbard AE. Super learner. *Statistical applications in genetics and molecular biology* 2007; **6**.

9. Westling T, van der Laan MJ, Carone M. Correcting an estimator of a multivariate monotone function with isotonic regression. *Electronic Journal of Statistics* 2020; **14**: 3032-69.

10. Chernozhukov V, Chetverikov D, Demirer M, Duflo E, Hansen C, Newey W, et al. Double/debiased machine learning for treatment and structural parameters. *The Econometrics Journal* 2018; **21**: C1-C68.
